# Navigating Parenting in Pediatric Oncology: Merging Psychodynamic Theory and Evidence-Based Practice

**DOI:** 10.3390/children12101395

**Published:** 2025-10-16

**Authors:** Yael L. E. Ankri, Amichai Ben-Ari

**Affiliations:** 1 Department of Psychology, Ariel University, Ariel 40700, Israel; amichaiba@ariel.ac.il; 2 Israel Center for Addiction and Mental Health (ICAMH), Hebrew University, Mount Scopus, Jerusalem 9190500, Israel

**Keywords:** pediatric oncology, parenting styles, psychodynamic theory, overprotection, winnicott, parental guilt, emotional resilience, good-enough parenting

## Abstract

**Highlights:**

**What are the main findings?**

- Parenting a child with cancer often leads to permissiveness and overprotection, which may hinder emotional resilience and coping.
- Integrating Winnicott’s psychodynamic theory with evidence-based practices helps to clarify how balancing parental responsiveness and structure supports healthier child outcomes during illness.

**What is the implication of the main finding?**

- A dialectical approach to parenting—balancing empathy and boundaries—is essential for fostering children’s emotional development in oncology settings.
- Health professionals can better support families by promoting “good-enough parenting,” helping parents manage guilt while encouraging the child’s autonomy and adaptive coping.

**Simple Summary:**

When a child is diagnosed with cancer, parents often struggle to know how best to support them emotionally. Many parents respond by being overly protective or giving in to every request, hoping to reduce the child’s distress. While this reaction is understandable, it can unintentionally make it harder for the child to cope with their illness and build emotional strength. This paper explores how parents can balance being caring and supportive with setting healthy limits, even during such a difficult time. Using the example of a young girl with leukemia and her family, we show how certain parenting responses, although well-meaning, can sometimes lead to more emotional and behavioral difficulties for the child. We also offer ways that therapists and healthcare teams can help parents find a more balanced approach. By showing love and empathy while also encouraging the child’s independence, parents can help their children face challenges with greater resilience. This approach not only supports the child’s emotional well-being during treatment, but also prepares them for life after illness.

**Abstract:**

Background/Objectives: Parenting a child with a chronic illness such as cancer presents distinct psychological challenges that often disrupt normative parenting patterns. Parents frequently struggle to maintain boundaries in response to their child’s heightened emotional needs, leading to overprotective or permissive behaviors. This study revisits Winnicott’s theory of the “good enough parent” and explores its application in the context of pediatric oncology. We aim to examine how a psychodynamic framework can be integrated with evidence-based practices to support parental functioning and promote child resilience during cancer treatment. Methods: This conceptual paper employs a qualitative, theory-driven case study approach. We analyze the case of a 6.5-year-old girl diagnosed with acute lymphoblastic leukemia (ALL), focusing on the evolving dynamics between the child’s regressive behaviors and the parents’ emotional responses. Winnicott’s developmental model is expanded to conceptualize parenting as a continuous balance between responsiveness and structure. Clinical dialogues illustrate the therapeutic process of guiding parents toward a more adaptive stance. Results: The analysis highlights how permissive parenting, driven by parental guilt and fear, may initially reduce child distress, but can inadvertently reinforce emotional dysregulation and dependency. The application of a dialectical interpretation of Winnicott’s theory allowed for a therapeutic shift, supporting parents in setting empathic yet firm boundaries. Conclusions: A balanced, dialectical approach to parenting—one that integrates emotional attunement with appropriate demands—can enhance a child’s psychological resilience during cancer treatment. Incorporating psychodynamic insights into clinical practice can help professionals guide families toward more adaptive, developmentally supportive caregiving strategies.

## 1. Introduction

Children facing chronic illnesses such as cancer often display turbulent emotional and behavioral challenges, leaving both their parents and professionals in the psycho-oncology field struggling to navigate these difficulties [1,2]. The intense demands of the sick Child—marked by anxiety, regression, and oppositional behaviors—can leave caregivers feeling helpless and uncertain about how to provide effective emotional support without becoming overly protective [3,4]. This article aims to return to foundational theories in developmental psychology to map and guide the parental path in the face of these challenges. By revisiting key theoretical concepts, we seek to offer insights for parents and professionals alike, helping them better understand the dynamics at play and how to foster resilience in the child.

Parenting is often conceptualized through three core dimensions: (1) *Parental responsiveness:* The adult adapts fully to the child’s immediate will (e.g., cancelling or postponing a scheduled blood draw until the child decides he or she is ready, even if ward routines are delayed). (2) *Parental demandingness*: The adult requires the child to meet an external expectation despite heightened emotional reaction (e.g., proceeding with the blood draw at the scheduled time, firmly holding position while the child protests). (3) *Dialectical parental balance:* The balance between overprotection and promoting autonomy [5]. These dimensions form the basis of Baumrind’s typology, a foundational framework in developmental psychology, categorizing parents as authoritative (high demandingness and responsiveness), authoritarian (high demandingness, low responsiveness), permissive (low demandingness, high responsiveness), or neglectful (low demandingness and responsiveness) [5,6]. While these parenting styles have been well-studied in general child development, parenting in the context of pediatric oncology introduces unique emotional and psychological challenges that disrupt typical parenting behaviors and exacerbate stress, guilt, and uncertainty.

Research in pediatric oncology has shown that authoritative parenting, characterized by high responsiveness and structured support, fosters resilience and self-regulation in children facing chronic illness [7,8]. However, parents of children undergoing cancer treatment often struggle to maintain boundaries, leading to overprotective behaviors akin to permissive parenting. This overprotection, while intended to shield the child from further distress, is linked to poorer emotional adjustment and increased dependency [4,9]. Children with cancer frequently experience heightened anxiety, depression, and behavioral issues, which are exacerbated by parenting styles that prioritize short-term emotional relief over long-term resilience [1,3,4,9,10]. Research in pediatric oncology has shown that permissive and overprotective parenting, often driven by parental guilt and fear, can hinder a child’s ability to develop adaptive coping mechanisms and emotional regulation, further complicating their psychological adjustment during treatment [2,11].

In the context of traumatic stress, our previous work [12] proposed a theoretical framework for understanding parenting, by reviewing Winnicott’s concept of the “good enough parent” [13].

Winnicott marks the main developmental goal of the baby as a transition from a state in which he is only aware of his internal reality to a state in which he can meet with the external reality and with other subjects. The Winnicottian theory can be schematically described as a three-stage developmental process that takes place within the “mother-baby dyad”. In the first stage, the newborn is in the state of “going on being”, and their mother has to be prepared to respond to all their needs from the position of the “preoccupied mother”. In the second stage, the baby begins to emerge from this inner reality; their mother, at this stage, should move to the position of the “good-enough mother”, which will allow for a response to the baby’s needs, but also some “empathic failures” that will allow the baby to recognize their limits. In the third and last stage, the child should reach the “ability to be alone”; their mother, at this stage, can enter a position where she presents herself as a separate subject [13,14,15].

The current paper aims to revisit and expand on Winnicott’s theory to better understand the concept of the “good enough parent” and its implications for parenting an ill child. We would like to propose two extensions to Winnicott’s theory, from the context of parenting w sick child.

**Figure 1:**
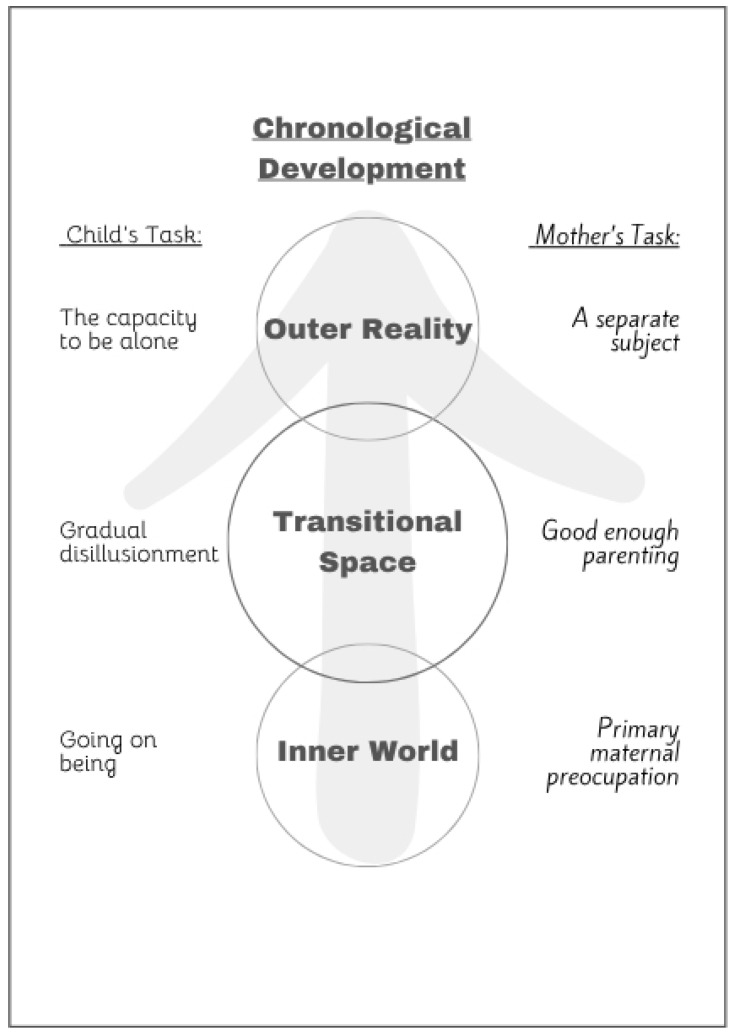
Original (chronological) Winnicottian model of parenting framework. The vertical axis depicts the child’s developmental progression from their inner world into the external reality through the transitional space. The gradual changes in maternal position enable them to progress from “Going on being” to “The capacity to be alone”.

**Figure 2:**
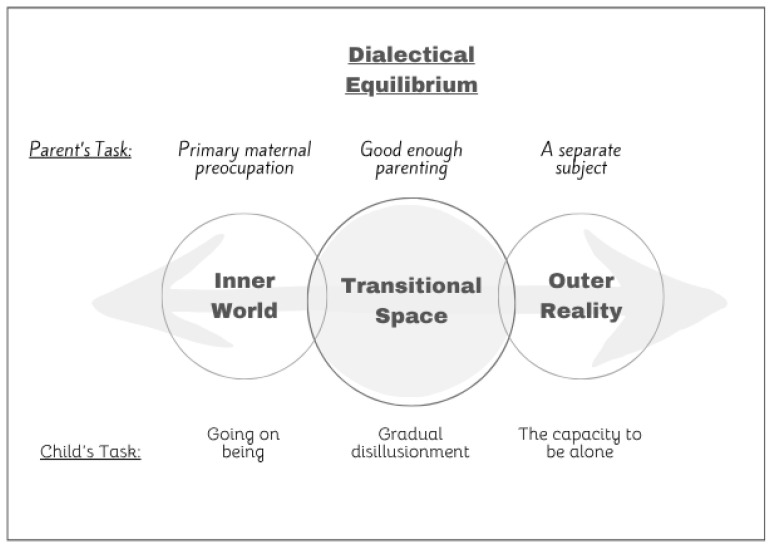
Expanded (Dialectical) Winnicottian parenting framework. The horizontal axis represents the ongoing tension between caregiver responsiveness (**left**) and caregiver demandingness (**right**). In this model, good-enough parenting is not a developmental stage, but rather a required equilibrium, enabling the child’s optimal frustration for ongoing growth and resilience.

The first expansion describes the three stages not as a developmental process (Figure 1), but as a continuous equilibrium that exists in every parent–child relationship, and also in every therapist–patient relationship (Figure 2).

The second extension indicates that many therapeutic theories tilted this balance in the direction of “preoccupied parenting” (left side, Figure 1) and that this bias is particularly strongly expressed in the context of treating a sick child. This expansion invites the public of therapists to return to a more balanced and dialectical position.

Our previous analysis will be applied to the pediatric oncology setting, examining how the interplay between responsiveness and demandingness impacts the psychological resilience of children undergoing cancer treatment. Through this lens, we aim to offer insights into how parents can navigate the psychological challenges posed by chronic illness while promoting their child’s emotional development and well-being.

### Clinical Example: Challenges in Parenting an Ill Child

Abigail, a 6.5-year-old girl, was hospitalized in the hematology-oncology ward after being diagnosed with acute lymphoblastic leukemia (ALL). Approximately two weeks into her hospitalization, her parents sought consultation with the ward psychologist. They reported that Abigail had met all developmental milestones on time and was functioning normally prior to her diagnosis. She attended school, played with friends, and was exploring her independence, as expected for her age. Abigail shared a close bond with her mother and maintained a healthy relationship with her father.

Following her diagnosis, Abigail’s initial reaction was marked by significant anxiety and withdrawal from her usual activities. She regressed, exhibiting behaviors such as nocturnal enuresis and using infantile language. Abigail frequently cried and demanded constant proximity to her parents. Over time, her use of her iPad initially bought to help distract her, became more frequent and intense. When they attempted to set limits on her screen time, particularly at bedtime, Abigail reacted with intense emotional outbursts, including crying, screaming, and aggression. She became insolent and oppositional, especially towards the medical team, refusing to cooperate with procedures unless she had access to her iPad. Empathizing with her distress and wanting to avoid exacerbating her anxiety, the parents relaxed their usual boundaries, allowing for more screen time in hopes it would provide comfort. As the weeks passed, Abigail’s behavior became increasingly demanding and brazen. She insisted on always using the iPad and resisted any attempts by her parents to curtail her usage. This permissive approach, initially intended to ease her suffering, seemed to fuel a cycle of escalating demands and deteriorating behavior. Concerned about the long-term effects of this pattern, the parents worried that Abigail’s dependency on the iPad and her aggressive reactions to limits were signs of deeper emotional struggles. Realizing the need for professional guidance, they decided to consult the ward psychologist.

In the two weeks leading up to that consultation, the ward routine had deteriorated sharply. Abigail refused physiotherapy sessions and even stopped sitting up for meals; her mother’s attempts to encourage participation were met with loud cries of “Don’t touch me!” and prolonged sobbing. Each evening, just before chemotherapy, Abigail demanded that the iPad stay with her during examinations; when a nurse declined, she hurled a pillow and struck her father’s arm. During the first session with the psychologist, both parents arrived visibly exhausted. The mother, tearful, described Abigail as “unrecognisable” and asked whether the therapist could “please fix her before she breaks completely.” The father, jaw clenched, added that every limit they tried to set “made things worse,” and he feared becoming the target of Abigail’s rage. Their joint plea was not for parenting guidance, but for an immediate remedy that would restore their daughter’s prior compliance.

This scenario illustrates the complex psychological dynamics at play in pediatric oncology. While permissive parenting may provide immediate relief to a child’s emotional distress, it can also undermine the development of essential coping mechanisms. Research in pediatric oncology highlights that overprotective or overly permissive behaviors, often borne out of parental guilt or fear, can hinder a child’s emotional resilience and adaptability [4,9].

## 2. Good-Enough or Preoccupied?

Winnicott’s revolutionary ideas centered around the concept of the transitional space, where a good-enough mother balances the inner and outer realities for her child’s gradual disillusionment. While Winnicott acknowledged the importance of both aspects of parenting, he insistingly cautioned against prematurely prioritizing the external world, which could lead to the development of a false self and trauma. Emphasizing a devoted, experience-close approach was revolutionary given the prevailing attitudes of the time, so it is not surprising that other theorists followed in Winnicott’s footsteps [16]. Many of them—such as Fromm (“symbiotic union”), Ogden (“mother-child dyad”), Tustin (“psychological birth”), Balint (“primary love”), and Kohut (“self-object”)—also emphasized the importance of the parents’ accommodations to the child’s inner world [17,18,19,20,21]. The socio-cultural context of his work may explain why Winnicott emphasized primary maternal preoccupation, but some interpretations of Winnicott and his followers misunderstood this as the sole aspect of good-enough parenting. Consequently, good-enough parenting is now often described as something closer to maternal preoccupation.

Despite numerous psychodynamic theories recognizing the child’s need for “optimal frustration” in the maturation process, many of them assume that this frustration will occur naturally, without the parent needing to push for change. According to Cohen and Lwow [22], most of these theories focus on the potential psychological damage to the child when forced to adapt prematurely to the adult world. Different theoreticians have different perspectives on this damage: Freud described these parents as castrating and accusatory [23,24], Klein portrayed them as oppressors [25], Winnicott illustrated an impinging and alienating parent, and Kohut presented such a parent as devaluing [21].

The conclusion is that to ensure children’s undamaged development, an environment must be created that does not expect them to adapt to their surroundings—in other words, an expectation-free environment. For generations of therapists, Winnicott, like many other theoreticians, emphasized the importance of leaning toward the child’s inner world and the crucial importance of the adapted maternal presence. In this regard, it is essential to mention that object relations theory, from which Winnicott hailed, emphasized the centrality of the correspondence between the therapist’s approach and the maternal position. Furthermore, this theory highlighted the importance of the therapist’s empathy skills and the accurate understanding of the patient’s inner world.

### Clinical Example: The Preoccupied Parent

In the therapy room, the mother, for the first time, expressed her profound guilt and deep-seated fears. She shared that she felt her daughter’s illness was a testament to her failure as a mother, unable to maintain her home, and that these feelings were paralyzing her. Additionally, she revealed her fear that Abigail hated her and would never forgive her. These thoughts and emotions surfaced during the therapeutic process, leading to important clarifications. The father also disclosed feelings of helplessness and detachment for the first time. He explained that he was trying his best to maintain some form of stability in the family, but the sorrow of his daughter and wife was overwhelming. He felt that everything he did only made things worse. He also admitted to feeling angry and disappointed with Abigail’s perceived ingratitude for everyone’s efforts.

At this point, the therapist realized that both parents were trapped in biased perceptions of their parental roles. The mother’s guilt and the father’s feelings of helplessness and frustration hindered their ability to address Abigail’s needs effectively.

The following sessions shifted attention from Abigail’s behavior to the parents’ inner world. The mother, speaking in a near-whisper, disclosed ruminative thoughts that she had “somehow caused the cancer” by missing an early check-up; these thoughts replayed at night and prevented her from sleeping more than two hours. She described compulsive scanning of Abigail’s face for any sign of pain, followed by an impulse to grant every request “before the suffering starts.”

The father voiced a contrasting but equally paralyzing experience: a sense of disqualification. “Nothing I do is right,” he said; attempts to enforce bedtime limits triggered Abigail’s rage and the mother’s reproachful look, leaving him oscillating between silent withdrawal and sudden outbursts of anger.

Together, these confessions revealed a dyad caught in primary parental pre-occupation—an over-absorption in the child’s moment-to-moment affect that eclipsed any long-term developmental perspective, precisely the imbalance described by Winnicott.

The medical staff also felt caught in a bind. Nurses noted that removing the iPad for vital-sign checks provoked meltdowns and parental complaints, so junior doctors began timing exams around screen use—an accommodation that quietly reinforced the cycle. As one senior nurse put it, “We’re caring for a tablet as much as a child.”

## 3. A Dialectical View of Good-Enough Parenting

As described earlier, Winnicott emphasized the importance of primary maternal preoccupation, which his followers sometimes understood as synonymous with good-enough parenting. However, Winnicott himself warned against the omnipotence illusion, which can arise when a mother struggles to adopt a new role as her child grows. Many other analytical authors have explored similar concepts. For instance, Kohut [26] introduced the idea of “optimal frustration,” Bion [27] discussed bearable deprivations as essential for the development of thinking, and Benjamin [28] highlighted the importance of the mother being an independent other with her own reactions, rather than just a mirror for the baby.

The following question remains: How should parents balance these seemingly opposite positions? A dialectical view was previously presented in the context of treating PTSD [12]. The role of the therapist in this context was to recognize the patient’s difficulty in discussing their trauma, providing validation and understanding while also emphasizing the importance of working through the trauma. This “dance” of the therapist—balancing between containing and validating the patient and encouraging and challenging them—illustrates a key aspect of Winnicott’s theory.

### 3.1. Clinical Example: The Dialectical Proposition

In a recent session, the therapist addressed the internal struggle of Abigail’s parents, stemming from their feelings of guilt and the need to compensate Abigail for the pain caused by her illness. The therapist adopted a Socratic approach to help the parents understand Abigail’s needs versus her wishes.

***Therapist:*** From what you describe, I understand that Abigail’s behavior worries you.

***Mom:*** Very true. I see her great distress and I don’t know what to do. I want to save her or at least reduce the amount of distress she is experiencing, but I feel helpless.

***Therapist:*** How else do you feel when Abigail yells at you and curses you?

***Mom:*** I feel guilty. I feel like I’m a bad mom and that my inability to stop her pain is indicative of my failure.

***Therapist:*** Are you saying that the role of a good parent is to prevent their child from feeling pain and distress?

***Mom:*** Yes, of course. This is what every parent wants.

***Therapist:*** You’re right, this is our natural aspiration as parents. But the question is, what makes a good parent when they can’t solve the child’s distress? What is their role in such a case?

***Mom:*** I really don’t know.

***Therapist:*** Let’s explore that. What do you think is the hidden message in your permissive reactions to Abigail’s escalating behavior? What are you telling her in your actions?

***Mom:*** I think the message is that we are here for her.

***Dad:*** That’s true, but we might also be conveying that she shouldn’t have to make any effort… And we’re even affirming that when she wants something, it means she really needs or deserves it.

***Mom:*** It’s hard to think that we might have nurtured her demanding side.

***Therapist:*** It’s understandable, and you obviously didn’t mean to. Often, our behavior can send a completely opposite message than what we intend. What do you think Abigail might be interpreting from your responses when you allow her to avoid any frustration?

***Mom:*** Maybe she thinks her pain is unbearable and that she can’t deal with it.

***Dad:*** And perhaps she feels there’s no need to face any frustration because we’ll always step in to fulfill her every wish.

***Therapist:*** That’s an important insight. The goal is to help Abigail build resilience. How can you convey to her that while her pain is indeed terrible and you are there to support her, she also has the strength to handle difficult feelings?

***Dad:*** We need to show her that we believe in her abilities, that she can manage her emotions even when it’s hard.

***Therapist:*** Exactly. It’s about setting limits that communicate not just “we will handle everything for you because you are weak” but also “this is difficult and painful, but we believe in your capacity to manage it.” How do you think you can start doing that?

***Mom:*** Maybe by setting small, achievable expectations and gradually increasing them, so she can see her progress and feel more capable.

***Dad:*** And by being consistent with our responses, showing her that we have faith in her ability to cope, even when we set limits.

***Therapist:*** That sounds like a strong plan. Remember, it’s about finding that balance where you support her without completely shielding her from the realities she needs to face.

Winnicott believed that embracing and managing internal conflicts and opposing psychological elements is crucial for a fuller, healthier life. A primary challenge for effective parenting is to find the “transitional space” and achieve “good-enough” parenting. This holistic approach acknowledges and respects the child’s personal experiences while understanding the traumatic impact of life’s harsh realities, such as abandonment and loss. Simultaneously, it gently pushes the child away from avoidance behaviors and integrates the realities of the external world.

This method emphasizes validating the individual’s personal experiences without becoming trapped by their subjectivity. Instead, it encourages engagement with the tangible world, fostering a balanced approach that recognizes both the internal and external realities. The dialectical perspective of good-enough parenting involves continuously navigating and reconciling these dual aspects, aiming for a harmonious development that supports both emotional resilience and realistic adaptation.

### 3.2. Clinical Example: Incermental Change

The therapist worked in small, tolerable steps. First, the parents agreed to insert a two-minute “pause ritual” before Abigail could reach for her iPad during routine blood draws. Session 1 of the trial ended in a full-blown tantrum and a 20 s compromise. By the fourth attempt Abigail managed 60 s, provided her mother counted aloud. She then reached the full two minutes in most procedures, and the ritual itself- counting, breathing, eye contact- became a predictable anchor. Nocturnal enuresis dropped from nightly to twice-weekly. Observing the trend, nurses felt confident to schedule vitals without negotiating screen use, and junior doctors shifted morning rounds back to their usual slot. Although setbacks still occurred, the progressive gains demonstrated that a deliberately modest boundary, held with warmth and consistency, could ripple outward: lowering the child’s reactivity, easing parental guilt, and restoring a semblance of routine to the ward.

## 4. Discussion: A Dialectical Approach to Good-Enough Parenting

The case of Abigail illustrates the psychological complexities that arise in pediatric oncology, not only for the child, but also for the parents. Abigail’s emotional regression and increased dependency reflect the broader mental health challenges faced by children with chronic illnesses, particularly when their usual coping mechanisms are disrupted by intensive medical treatments. The permissive approach taken by Abigail’s parents, though initially well-intended, resulted in a pattern of escalating demands and emotional volatility, revealing how overprotection can undermine a child’s ability to develop adaptive coping strategies during treatment.

Research in pediatric oncology consistently shows that parental overprotection, often triggered by guilt and fear, can interfere with a child’s capacity to develop resilience in the face of illness [4]. Abigail’s increasing dependency on her iPad and her inability to tolerate frustration align with findings that overpermissiveness, while providing immediate emotional relief, fosters long-term emotional dysregulation and increased anxiety [9]. Our case supports this, as Abigail’s behaviors became more oppositional over time, reflecting her struggle to cope with the stress of her illness and the lack of consistent boundaries.

This case advances current understanding by providing an in-depth look at how parenting styles impact a child’s mental health during cancer treatment, a theme that has been explored in Psycho-Oncology. Previous studies, such as Ernst et al. [11], have highlighted that parenting stress is heightened in the context of pediatric cancer, often leading to shifts in parenting behaviors. However, this case expands on those findings by specifically examining how permissiveness and avoidance of setting limits in response to a child’s distress can complicate the treatment process and hinder emotional growth. Research consistently shows that even children facing serious illnesses benefit from clear boundaries, as they provide a sense of security, structure, and predictability during an otherwise chaotic time [29,30,31]. Studies in pediatric psychology emphasize that appropriate boundaries can help children develop emotional resilience and coping strategies by encouraging them to confront and manage their distress rather than avoid it [3,31]. Moreover, permissiveness in response to illness may lead to greater emotional dysregulation and anxiety, ultimately hampering long-term psychological adaptation [1]. Unlike traditional research that focuses primarily on the emotional effects on the child, our analysis shows how these parenting patterns can interfere with a child’s emotional development and adjustment to treatment over time.

Elements of our dialectical parenting stance are already embedded, often implicitly, in several empirically supported programs for families facing childhood cancer. Examples can be found in the empathic listening and emotional-labelling skills taught in “Bright IDEAS”, a problem-solving program that encourages parents to pause, acknowledge the child’s feeling, and validate distress before generating solutions [32]; the structure-setting techniques used in “COPE” (Creating Opportunities for Parent Empowerment), where caregivers practice delivering clear, developmentally scaled expectations during medical procedures [33]; and in “SCCIP-Plus”, a trauma-focused cognitive-behavioural family therapy, pairing supportive dialogue with graded exposure to cancer-related stressors [34]. Our contribution is to make this integration explicit and theoretically grounded, providing a shared language that links psychodynamic ideas (e.g., optimal frustration) to the concrete skills clinicians already employ. Future research can test whether framing existing interventions within this dialectical rubric enhances parent engagement and child outcomes.

Furthermore, our application of Winnicott’s concept of the “good-enough parent” emphasizes the need for parents to strike a balance between responsiveness and structure, even under the extraordinary circumstances of pediatric oncology. Winnicott [35] argued that children must experience “optimal frustration” to develop coping skills, and our case demonstrates how shielding a child from all frustration can prevent the development of critical emotional resilience. This dialectical view of parenting, which stresses the balance between supporting a child’s emotional needs and encouraging autonomy, aligns with current psychological interventions aimed at helping parents navigate the psychosocial challenges of cancer treatment [22,36,37,38,39].

In summary, this discussion highlights how permissive parenting in the oncology context can lead to negative psychological outcomes for children, such as emotional dependence and difficulty managing distress. By integrating Winnicott’s framework, we offer a theoretical and practical lens for understanding how parents can support their children’s mental health through both emotional validation and structured expectations. Future research should further explore the long-term effects of different parenting styles in pediatric oncology to provide evidence-based guidelines for interventions that can promote both emotional and psychological resilience in children facing chronic illnesses.

1. **Cultural Considerations**

Expectations for “good-enough” parenting vary cross-culturally. In more collectivist contexts, autonomy-promoting demandingness may be expressed through family-centred obligations (e.g., cooperating with procedures “for the good of the group”), whereas responsiveness often emphasizes relational harmony rather than individual emotion-labelling [40,41]. Our dialectical stance is therefore culturally flexible: the clinician gauges which behaviors signal warmth and which signal structure *in that family’s cultural script* and then balances them intentionally. Future trials should stratify samples by cultural orientation to test whether the same balance point yields comparable child outcomes across cultures.

2. **Limitations**

The article relies on a single illustrative case; therefore, conclusions should be viewed as analytic rather than statistical generalizations. Additional cases, prospective cohort studies, and mixed-methods trials are needed to test whether the dialectical balance predicts child resilience across diagnoses, phases of treatment, and healthcare systems. Embedding brief repeated-measures (e.g., PIP, Child Anxiety) within such studies would permit quantitative validation, while qualitative interviewing could capture contextual nuances that a single vignette cannot provide.

## 5. Conclusions

In conclusion, the dialectical approach to parenting, which combines responsiveness and demandingness, is critical for fostering the emotional and psychological well-being of children, particularly those facing significant health challenges like cancer. By providing both emotional support and a structured environment, parents can help their children develop resilience, self-regulation, and the necessary skills to navigate life’s difficulties. This balanced parenting style, reflective of Winnicott’s good-enough parenting, not only benefits sick children, but also emphasizes the importance of such an approach in the development of healthy children. It highlights how warmth, support, structure, and discipline collectively contribute to a child’s overall well-being and growth. (Box 1)

Box 1 **Practical Guidance for Clinicians.**

- **Validate first, then set one clear limit**. Empathically name the child’s feeling before introducing a brief, developmentally appropriate demand.
- **Use micro-doses of frustration**. Start with a 30-s delay or one physiotherapy repetition; lengthen only after success.
- **Narrate the dialectic aloud**. Say, “I see you’re scared *and* we can do this together.”
- **Debrief with staff**. Align nurse and physician scripts so the same limit is upheld across shifts.

For the medical team, it is essential to adopt a dialectical approach that simultaneously validates the child’s emotional experiences while encouraging them to confront the realities of their illness and treatment. Medical professionals should aim to create a space where a child’s fears, anxieties, and frustrations are acknowledged and respected, offering empathy and support in times of distress. At the same time, they must guide both the child and the parents to face necessary challenges—such as medical procedures and the establishment of appropriate boundaries—that foster emotional resilience. By striking this balance, the medical team can help the child develop a sense of autonomy and competence, which are critical for long-term psychological adaptation. Encouraging parents to set boundaries, even when difficult, and to hold a vision of their child’s ability to cope, will support a more adaptive and resilient response to the stressors of chronic illness.

This paper has explored the complexities and nuances of good-enough parenting, particularly in the context of children with chronic illnesses such as cancer. By integrating Winnicott’s theories with contemporary understandings of parental responsiveness and demandingness, we have underscored the importance of a balanced, dialectical approach to parenting. This approach not only supports the immediate emotional needs of the child, but also fosters long-term resilience and development, preparing them to face life’s inevitable challenges with confidence and capability.

## Data Availability

The original contributions presented in this study are included in the article. Further inquiries can be directed to the corresponding authors.
